# A necessary criterion for obtaining accurate lattice parameters by Rietveld method

**DOI:** 10.1038/s41598-017-15766-y

**Published:** 2017-11-13

**Authors:** Masami Tsubota, Jiro Kitagawa

**Affiliations:** 1 Physonit Inc., 6-10 Minami-horikawa, Kaita, Aki, Hiroshima, 736-0044 Japan; 2 Department of Electrical Engineering, Faculty of Engineering, Fukuoka Institute of Technology, 3-30-1 Wajiro-higashi, Higashi-ku, Fukuoka, 811-0295 Japan

## Abstract

To obtain the lattice parameters accurately by the Rietveld method, the relationship between the lattice parameters and the peak-shift, which is the deviation in diffraction angle from the theoretical Bragg position, was studied. We show that the fitting accuracy of lattice parameters is related directly to the well reproducibility of the peak-shift. This study unveils that the peak-shift consists of the experimental and the analytical ones. The analytical peak-shift erroneously lowers a reliability factor *R*_wp_, which has, so far, been the conventional criterion of fit. The conventional Rietveld method obtains a unit-cell which is a homothetic (proportional) unit-cell of the true one. We propose an additional criterion based on the peak-shift to obtain the true lattice parameters accurately. Our criterion can achieve reproducibility reasonably well for the experimental peak-shift, leading to highly improved accuracy of the lattice parameters.

## Introduction

Structural study for powder materials relies on the Rietveld method, which is capable of refining the structural and magnetic parameters from diffraction data^1–3^. However, it is fundamentally difficult to determine accurately the refinement parameters^4–7^. In the Rietveld method, the weighted sum of squares residual, *S*_r_, between the observed and the calculated intensities of powder diffraction data is minimized in a nonlinear least-squares method. The calculated intensity includes the peak-shift that is absolutely inevitable in the experiment. To evaluate quantitatively the best fit of the data, several reliability-factors such as *R*_wp_, *R*_p_, *R*_e_, *R*_*F*_, *S* and *χ* ^2^ are proposed^3,4^. The most accepted factor is the weighted-profile *R*, termed as *R*_wp_, where the numerator includes *S*_r_ that is minimized during the refinements. The goodness-of-fit, *S* or *χ* ^2^ ≡ *S* ^2^, is used as another useful numerical criterion^4^. The *S*-value of 1.3 or less is empirically considered to be satisfactory. However, a poor counting statistics or a high background also makes *S* smaller; the *S*-value sometimes turns out to be less than 1.0. On the other hand, *S* may possibly be larger than 1.3 even for the best fitting with an appropriate model. Young and co-workers have suggested that these values to be given in publication^8^.

Strictly speaking, there is no general agreement on these criteria in the Rietveld method. Other studies have concluded that viewing the profile-plots is more effective than *R*-values to determine the quality of a refinement^5–7^. As such, the refined structural parameters have been found to differ from researcher to researcher. For instance, Hill summarized the results of Rietveld refinements on the project undertaken by the Commission on Powder Diffraction of the International Union of Crystallography^7^. Several specialists analysed the *standard* PbSO_4_ powder diffraction pattern, measured by a conventional Bragg–Brentano diffractometer using Cu *Kα* radiation. The lattice parameters *a*, *b* and *c* are in the range of 8.4764–8.4859 Å, 5.3962–5.4024 Å and 6.9568–6.9650 Å, respectively. The accuracy of the lattice parameters is of an order of 0.01 Å (=10 × 10^−3^ Å), which is incomparably large considering that the linear thermal expansion coefficient is of an order of 10^−5^ K^−1^ to 10^−6^ K^−1^ for general solid materials^9^. Furthermore, the weighted mean parameters for *a*-, *b*- and *c*-axes are 8.4804(4) Å, 5.3989(3) Å and 6.9605(2) Å, respectively. They are in good agreement with those determined from single-crystal X-ray diffraction data^7,10^ which is generally accepted to be high-accuracy. These facts mean that either smaller or larger lattice parameter compared to the true one is possibly obtained depending on a researcher by the Rietveld method. This is a critical disadvantage to study the dependences of lattice parameters on temperature, composition, pressure and so on. A technique to determine refinement parameters accurately is needed for the Rietveld method.

We shed light on the peak-shift that tends to be overlooked. This paper proposes an additional criterion, focusing on a fitting accuracy along the horizontal-axis of powder diffraction data, to determine the lattice parameters accurately. In the following, we demonstrate that our criterion enables the well reproducibility of the peak-shift, leading to highly improved accuracy of the lattice parameter by two or more digits lower compared to that obtained by the conventional Rietveld method.

The X-ray diffraction pattern of standard reference material (SRM) 660a (lanthanum hexaboride)^11^ from the National Institute of Standards and Technology (NIST) collected with Cu *K*α_1_ radiation was used in this study. We focused on the maximum diffraction angle (2*θ*_max_) of the data used in the analysis. We conducted several conventional Rietveld refinements in the 2*θ*-range from 18° to 2*θ*_max_, where 2*θ*_max_ was in between 52° and 152°. There were five Bragg-peaks for 2*θ*_max_ = 52° and twenty-four Bragg-peaks for 2*θ*_max_ = 152°. The representative results for 2*θ*_max_ = 152° and 92° are demonstrated.

## Results

### Rietveld refinements

In the conventional Rietveld refinement, the lattice parameters are *a* ^cnv,(152)^ = 4.15655(1) Å with *R*_wp_^cnv,(152)^ = 8.203% and *a* ^cnv,(92)^ = 4.15811(22) Å with *R*_wp_^cnv,(92)^ = 8.610%, where the superscripts ‘cnv’, (152) and (92) refer to the “*conventional*”, 2*θ*_max_ = 152° and 92°, respectively. Here, *a* ^cnv,(152)^ and *a* ^cnv,(92)^ are 0.37 × 10^−3^ Å (or 0.0089%) smaller and 1.19 × 10^−3^ Å (or 0.0286%) larger than *a*_SRM_ ≃ 4.15692(1) Å, respectively^11^. The Rietveld refinements with a fixed value of *a*_SRM_ were conducted. The reliability factors *R*_wp_^fix,(152)^ and *R*_wp_^fix,(92)^ are 8.355% and 8.623%, respectively, where the superscript ‘fix’ refers to the *“fixed”*. Significantly, *R*_wp_^fix^ is larger than *R*_wp_^cnv^, implying that *R*_wp_ is an incomplete criterion of fit. Note that a difference between *R*_wp_^fix^ and *R*_wp_^cnv^ is not caused by the difference of the number of parameters in each refinement because *R*_e_, which corresponds to mathematically expected *R*_wp_, is *R*_e_^fix,(152)^ = *R*_e_^cnv,(152)^ = 8.203% and *R*_e_^fix,(92)^ = *R*_e_^cnv,(92)^ = 4.090%, and are the same with each other independent on the number of parameters.

**Figure 1:**
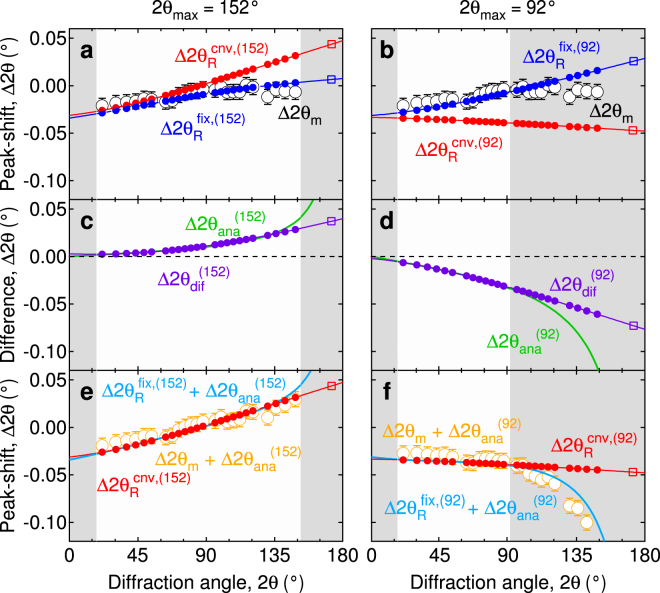
Diffraction angle dependence of the peak-shifts. (**a,b**) Δ2*θ*_R_^cnv^, Δ2*θ*_R_^fix^ and Δ2*θ*_m_. Δ2*θ*_R_^cnv^ is obtained by conventional Rietveld refinement. Lattice parameter is fixed at *a*_SRM_ in Rietveld refinement for Δ2*θ*_R_^fix^. Δ2*θ*_m_ is manually calculated by comparing 2*θ*-angle values of Bragg-peaks in the raw data and certification by NIST. (**c,d)** Difference Δ2*θ*_dif_ ≡ Δ2*θ*_R_^cnv^ − Δ2*θ*_R_^fix^ and analytical peak-shift Δ2*θ*_ana_. Horizontal dash line with zero intensity is drawn as visual guide. (**e,f)** Sums Δ2*θ*_m_ + Δ2*θ*_ana_ and Δ2*θ*_R_^fix^ + Δ2*θ*_ana_. Δ2*θ*_R_^cnv^ is again plotted for comparison. For all the panels, open squares at approximately 2*θ* = 172° are the calculated data for a reflection with the Miller indices of 432 and 520 that is not observed in the data. Note that the 2*θ*-regions with grey background are not used in the Rietveld refinement. Left and right panels are for 2*θ*_max_ = 152° and 92°, respectively. The error bars indicate the measurement error of the diffraction data in (**a**,**b**,**e)** and (**f)**.

Figure 1a and b show the 2*θ*-dependence of the peak-shift Δ2*θ*_R_ computed with the following equation^12^:

1 $${\rm{\Delta }}2{\theta }_{{\rm{R}}}=Z+{D}_{{\rm{s}}}\,\cos \,\theta +{T}_{{\rm{s}}}\,\sin \,2\theta ,$$

where *Z* is the zero-point shift (also known as the zero error), *D*_s_ the specimen-displacement parameter and *T*_s_ the specimen-transparency parameter. Manually estimated peak-shift, Δ2*θ*_m_, is also plotted. Note that the 2*θ*-regions with grey background in Fig. 1 are not used in the Rietveld refinement. Clearly, Δ2*θ*_R_^fix^ and Δ2*θ*_m_ correspond well with each other within an error bar in the analysis 2*θ*-region (white area). In contrast, Δ2*θ*_R_^cnv^ differs from Δ2*θ*_R_^fix^ and Δ2*θ*_m_ especially in the large 2*θ*-region.

Figure 1c and d show the 2*θ*-dependence of the difference, Δ2*θ*_dif_ ≡ Δ2*θ*_R_^cnv^ − Δ2*θ*_R_^fix^, which could be zero when *a* = *a*_SRM_. Otherwise, the absolute value of Δ2*θ*_dif_ increases with 2*θ*. Moreover, Δ2*θ*_dif_ is not negligible with respect to the magnitude compared to Δ2θ_R_^fix^ and Δ2*θ*_m_ (Fig. 1a and b). Note that Δ2*θ*_dif_ can be expressed by Eq. (1) with a different set of values of (*Z* ^cnv^, *D*_s_^cnv^, *T*_s_^cnv^) and (*Z* ^fix^, *D*_s_^fix^, *T*_s_^fix^). Most importantly, in the analysis 2*θ*-range, the 2*θ*-dependence of Δ2*θ*_dif_ corresponds well with that of Δ2*θ*_ana_, which is expressed as:

2 $${\rm{\Delta }}2{\theta }_{{\rm{ana}}}=2\{{\sin }^{-1}(\frac{1}{A}\,\sin \,\theta )-\theta \},$$

where *A* is the proportional coefficient. Here, Eq. (2) is not obtained by fitting the experimental data but is formulated by rearranging the following two Bragg’s equations and, therefore holds for any crystal system:

$$\begin{array}{c}2d\,\sin \,\frac{2\theta }{2}=\lambda ,\\ 2(Ad)\,\sin \,\frac{2\theta +{\rm{\Delta }}2{\theta }_{{\rm{ana}}}}{2}=\lambda .\end{array}$$

The coefficients *A* ^cnv,(152)^ and *A* ^cnv,(92)^ are *a* ^cnv,(152)^/*a*_SRM_ = 0.999911 and *a* ^cnv,(92)^/*a*_SRM_ = 1.000286, respectively. Equally important is that Δ2*θ*_m_ + Δ2*θ*_ana_ as well as Δ2*θ*_R_^fix^ + Δ2*θ*_ana_ are in good agreement with Δ2*θ*_R_^cnv^ in the analysis 2*θ*-range and enhance against Δ2*θ*_R_^cnv^ beyond 2*θ*_max_ (Fig. 1e and f).

### Criteria of fit

**Figure 2:**
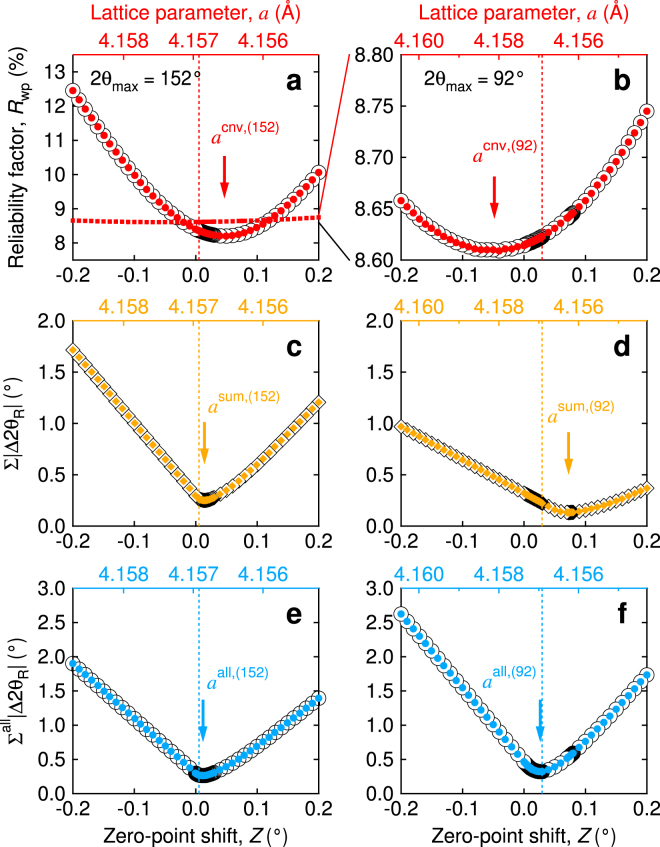
Conventional and candidate criteria of fit. *Z*- and *a*-dependences of (**a)**, *R*_wp_^fix^ for 2*θ*_max_ = 152°, (**b)**, *R*_wp_^fix^ for 2*θ*_max_ = 92°, (**c)**, Σ|Δ2*θ*_R_| for 2*θ*_max_ = 152°, (**d)**, Σ|Δ2*θ*_R_| for 2*θ*_max_ = 92°, (**e)**, Σ^all^|Δ2*θ*_R_| for 2*θ*_max_ = 152°, (**f)**, Σ^all^|Δ2*θ*_R_| for 2*θ*_max_ = 92°. Vertical dot line indicates *a* = *a*_SRM_. The minimum for each criterion is shown by the arrow. (**a)**, *R*_wp_^fix^ for 2*θ*_max_ = 92° is also plotted for comparison (small dots). Note that relationship between *Z* and *a* for 2*θ*_max_ = 152° (left panels) and that for 2*θ*_max_ = 92° (right panels) are not the same.

To investigate a criterion of fit in detail and study how the peak-shift affects the result, we have conducted several Rietveld refinements with a fixed value of *Z*. Figure 2 shows the *Z*- and *a*-dependences in the conventional criterion as well as by the criteria set in this study. The sums are carried out over all the Bragg-peaks in the analysis 2*θ*-range for Σ|Δ2*θ*_R_| and the whole 2*θ*-range for Σ^all^|Δ2*θ*_R_|. Note that Σ|Δ2*θ*_R_| and Σ^all^|Δ2*θ*_R_| are calculated from the result after the refinement. The convergence in the refinement is judged by using *R*_wp_. For Σ|Δ2*θ*_R_|, the number of Bragg-peaks in the sum depends on 2*θ*_max_, and is 24 for 2*θ*_max_ = 152° and 13 for 2*θ*_max_ = 92°. In contrast, the number of Bragg-peaks is always 25 for Σ^all^|Δ2*θ*_R_|, including a reflection with the Miller indices of 432 and 520 (lattice spacing *d* ≃ 0.772 Å) at 2*θ* ≃ 172° that is measureable in principle but is not observed in the data.

The conventional criterion *R*_wp_ shows a parabolic curve with the minimum values of 8.203% at *a* ^cnv,(152)^ = 4.15655(1) Å and 8.610% at *a* ^cnv,(92)^ = 4.15811(22) Å as shown in Fig. 2a and b. Importantly, the minimum of *R*_wp_ is not at *a*_SRM_, which is a strong evident that *R*_wp_ itself is an insufficient criterion to obtain the true lattice parameter. Further, the range of *R*_wp_ for 2*θ*_max_ = 92° is much smaller than that for 2*θ*_max_ = 152°. It suggests that for the smaller 2*θ*_max_, it is more difficult to distinguish the minimum *R*_wp_ correctly.

A potential criteria Σ|Δ2*θ*_R_| shows a V-shaped curve with the minimum values at *a* ^sum,(152)^ = 4.15684(0) Å and *a* ^sum,(92)^ = 4.15625(2) Å, where the superscript ‘sum’ refers to the *“sum”* of the peak-shift (Fig. 2c and d). The lattice parameter *a* ^sum^ is closer to *a*_SRM_ compared with *a* ^cnv^. The magnitude of Σ|Δ2*θ*_R_| for 2*θ*_max_ = 92° is smaller than that for 2*θ*_max_ = 152°, which is reasonable considering the number of Bragg-peaks in the sum. Our proposed criterion Σ^all^|Δ2*θ*_R_| shows a sharper V-shaped curve than Σ|Δ2*θ*_R_| with the minimum values at *a* ^all,(152)^ = 4.15686(0) Å and *a* ^all,(92)^ = 4.15696(2) Å, where the superscript ‘all’ refers to the sum of *“all”* values of Δ2*θ*_R_ (Fig. 2e and f). The lattice parameter *a* ^all^ is much closer to *a*_SRM_ compared with *a* ^cnv^ and *a* ^sum^. With decreasing 2*θ*_max_, the magnitude of Σ^all^|Δ2*θ*_R_| increases and the V-shape becomes sharper.

**Figure 3:**
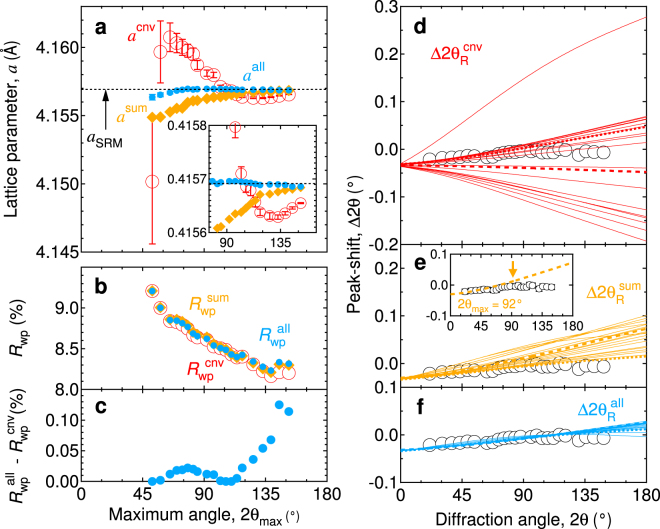
Comparison of obtained lattice parameters and peak-shifts. (**a)**, 2*θ*_max_-dependence of lattice parameters *a* ^cnv^, *a* ^sum^ and *a* ^all^. The error bars represent the standard error *σ* in the Rietveld refinement. Horizontal dot line indicates *a*_SRM_. Inset: Enlarged view of *a* in the range of 75° ≤ 2*θ*_max_ ≤ 165°. (**b)**, 2*θ*_max_-dependence of reliability factors *R*_wp_^cnv^, *R*_wp_^sum^ and *R*_wp_^all^. **c**, 2*θ*_max_-dependence of difference *R*_wp_^all^ − *R*_wp_^cnv^. (**d,e,f)**, 2*θ*-dependences of peak-shifts Δ2*θ*_R_^cnv^, Δ2*θ*_R_^sum^ and Δ2*θ*_R_^all^. Bold dot lines and dash lines are for 2*θ*_max_ = 152° and 92°, respectively. (**e)**, Inset: Enlarged view of Δ2*θ*_R_^sum^ for 2*θ*_max_ = 92°. Δ2*θ*_R_^sum^ starts to deviate at approximately 2*θ*_max_ as shown by the arrow with increasing 2*θ*.

Figure 3a demonstrates the 2*θ*_max_-dependence of the lattice parameters obtained by several criteria. First, *a* ^cnv^, which is obtained by the conventional Rietveld method, shows a large deviation from *a*_SRM_ and strong dependence on 2*θ*_max_. The maximum deviation from *a*_SRM_ is >10 × 10^−3^ Å, which is in the same order as that in Hill’s report^7^. Next, *a* ^sum^, which is determined with the minimum of Σ|Δ2*θ*_R_|, approaches toward *a*_SRM_ with increasing 2*θ*_max_. The smallest deviation from *a*_SRM_ is 0.08 × 10^−3^ Å at 2*θ*_max_ = 152°. Subsequently, *a* ^all^, which is determined by using Σ^all^|Δ2*θ*_R_|, corresponds well with *a*_SRM_ even for the smaller 2*θ*_max_. The deviation from *a*_SRM_ is 0.60 × 10^−3^ Å at the most and within 0.06 × 10^−3^ Å above 2*θ*_max_ = 74°. The accuracy is improved by two or more orders of magnitude compared with that of the conventional Rietveld method.

Figure 3b shows the 2*θ*_max_-dependence of *R*_wp_’s. It is clear that *R*_wp_ increases with decreasing 2*θ*_max_. For all 2*θ*_max_, the values of *R*_wp_^cnv^ are smaller than those of *R*_wp_^all^ despite the fact that *a* ^cnv^ does not correspond to *a*_SRM_. The difference, *R*_wp_^all^ − *R*_wp_^cnv^, becomes smaller with decreasing 2*θ*_max_ and is 0.02% or less below 2*θ*_max_ = 120° as shown in Fig. 3c. It becomes zero at some 2*θ*_max_’s, implying the impossibility in distinguishing the true solution exclusively by the *R*_wp_-value.

Figure 3d–f show the peak-shifts determined with the minima of *R*_wp_, Σ|Δ2*θ*_R_| and Σ^all^|Δ2*θ*_R_|. Clearly, Δ2*θ*_R_^cnv^ does not reproduce Δ2*θ*_m_, reflecting a mismatch of the lattice parameter between *a* ^cnv^ and *a*_SRM_. Although Δ2*θ*_R_^sum^ is closer to Δ2*θ*_m_ than Δ2*θ*_R_^cnv^, it deviates from Δ2*θ*_m_ above 2*θ*_max_ as shown in the inset of Fig. 3e as an example. Additionally, Δ2*θ*_R_^all^ well reproduces Δ2*θ*_m_ for the all 2*θ*_max_ (Fig. 3f). These facts indicate that the fitting accuracy relates directly to the well reproducibility of the peak-shift.

## Discussion

The present study reveals several critical findings. Firstly, the 2*θ*-dependence of peak-shift does not obey Eq. (1) in the calculation; instead follows the equation:

3 $${\rm{\Delta }}2{\theta }_{{\rm{R}}}=\zeta +{\delta }_{{\rm{s}}}\,\cos \,\theta +{\tau }_{{\rm{s}}}\,\sin \,2\theta +2\{{\sin }^{-1}(\frac{1}{A}\,\sin \,\theta )-\theta \},$$

where *ζ* is the zero-point shift, *δ*_s_ the specimen-displacement parameter, *τ*_s_ the specimen-transparency parameter and *A* the proportional coefficient to lattice spacing (Fig. 1e and f). Equation (3) holds for any crystal system and can be simply rewritten as:

4 $${\rm{\Delta }}2{\theta }_{{\rm{R}}}={\rm{\Delta }}2{\theta }_{\exp }+{\rm{\Delta }}2{\theta }_{{\rm{ana}}},$$

where Δ2*θ*_exp_ is the experimental peak-shift by the geometry (includes design-geometry of instrument as well as specimen-geometry) and Δ2*θ*_ana_ is the analytical peak-shift caused by the mismatch of the lattice parameters. Notably, Δ2*θ*_ana_ exists in the calculation only when *A* ≠ 1. Considering Eqs (3) and/or (4), one cannot obtain the true peak-shift when Δ2*θ*_ana_ ≠ 0 (*A* ≠ 1). Secondly, Δ2*θ*_ana_ can be fitted very well by Eq. (1) in the analysis 2*θ*-range (Fig. 1c and d). The finite value of Δ2*θ*_ana_, therefore, induces a false peak-shift with irrelevant lower-*R*_wp_ (Fig. 2a and b). As a result, a homothetic unit-cell, which is proportional to the true one, is obtained in the conventional Rietveld method. To obtain the correct unit-cell, Δ2*θ*_ana_ = 0 should be imposed. Finally, we have proposed an additional criterion, Σ^all^|Δ2*θ*_R_|, which measures the fitting accuracy along the horizontal-axis of the diffraction data and is capable of preventing Δ2*θ*_ana_ from enhancing. By combining our criterion with *R*_wp_, we can well reproduce the peak-shift (Fig. 3f). Consequently, we can determine the lattice parameter within the accuracy of 0.06 × 10^−3^ Å (Fig. 3a). Incidentally, we deduce that there was no need to consider too much detail about the peak-shift in the early stage of developing the method because the angle-dispersive neutron data was used^3,13^. Neutron has high transparency against the materials. Enough high-angle data, *e.g*. 2*θ*_max_ = 144° (ref.^3^), with quite broad Bragg-peaks were generally obtained using an old-fashioned reactor source. As a result, the 2*θ*-dependence of peak-shift was approximately constant and could easily be reproduced. In fact, Rietveld applied a zero-shift parameter as the peak-shift function which is independent on 2*θ* (ref.^3^).

Our present findings possibly accelerate designing novel materials since a comparative study between the experiment and theory^14^ may be achieved with high-accuracy. The criterion we set in this report would be applicable for structure determination from powder diffraction^15–17^ including indexing the diffraction peaks^18–20^ and the profile decomposition^21–23^ as well as a quality management of mass production of materials in industry. Further study related to the structural parameters in the unit-cell is desirable.

In summary, an additional criterion, Σ^all^|Δ2*θ*_R_|, to determine accurately the lattice parameter by the Rietveld method from powder diffraction data has been proposed. The refinements of the same data with different fixed-values of peak-shift parameter lead to different values of reliability factor, *R*_wp_. The refined lattice parameter at the minimum *R*_wp_-value is different from the correct one. The peak-shift includes the analytical Δ2*θ*_ana_ as well as the experimental Δ2*θ*_exp_ in the calculation. Δ2*θ*_ana_ must be neutralized for the analysis because it results in a false unit-cell that is proportional to the true one with the incorrect lower *R*_wp_-value when Δ2*θ*_ana_ ≠ 0. Our criterion allows well reproducibility of the peak-shift through the highly accurate determination of lattice parameter by two or more digits lower than that compared with the conventional Rietveld method.

## Methods

### Powder diffraction data

Diffraction pattern used in this study was measured by Le Bail and distributed on the website^24^. The data file with a name of “660a-2.dat” in a compressed file, x-celerator.zip, was used. The X-ray diffraction pattern for SRM 660a^11^ was carefully collected in the range of 2*θ* = 18.003°−151.995° with a step of 0.008° by using a conventional diffractometer (Philips X’Pert, equipped with an X’Celerator detector) with Cu *Kα*_1_ radiation. Twenty-four diffraction peaks were observed. Total measurement time was more than 17 h and the largest intensity was more than 100000 counts, realizing very good statistics and a high signal-to-background ratio. The diffraction peaks were very sharp as a full width at half-maximum (FWHM) of a diffraction peak were approximately 0.03° at the lowest angle and 0.17° at the highest angle. The peaks were fairly symmetric for the in-house data. The lattice parameter *a*_SRM_ = 4.1569162(97) Å ≃ 4.15692(1) Å at 22.5 °C has been clarified by NIST^11^.

### Data analysis approach

#### Rietveld refinements

The Rietveld program RIETAN-FP^25^ was selected to analyse the data in this study. Taking the rounding error of the program into consideration, a value of the wavelength *λ* (1.540593 Å for Cu *Kα*_1_ radiation) in RIETAN-FP is the same as that used in the computation for SRM 660a^11^ by NIST (*λ* = 1.5405929(5) Å)^26^. Note that the other major Rietveld programs use a slightly different value for Cu *Kα* radiation as default. For example, in GSAS^27^, GSAS-II^28^, FullProf^29^, Z-Rietveld^30^ and TOPAS^31^, the wavelengths of Cu *Kα*_1_ are 1.5405 Å, 1.54051 Å, 1.54056 Å, 1.54056 Å and 1.540596 Å, respectively. The profile function of a Thompson-Cox-Hastings pseudo-Voigt function^32^ was used. Howard’s method^33^, which is based on the multi-term Simpson’s rule integration, was employed for the profile asymmetry. Profile cut-off was 0.001%. The background function was the sixth order of Legendre polynomials.

In addition to the conventional Rietveld refinement, several sets of Rietveld refinement, with different fixed-values for the first term *Z* of the peak-shift, were performed. Here, the other refinement parameters were refined. This is because we have assumed that a parameter *Z*, which is different from the true value *Z* ^true^, gives *R*_wp_ larger than that for the true value *R*_wp_^true^. The range of *Z* between −0.2° and 0.2° was chosen considering a FWHM of a diffraction peak. For each set, a total of 157 calculation-steps were conducted to confirm that our procedure was enough to converge. Thus, our calculation is the fixed routine one that is applied to the same data set starting from different fixed *Z*-values for Rietveld refinement.

#### Peak-shift estimation

The peak-shift Δ2*θ*_m_ ≡ 2*θ*_SRM_ − 2*θ*_obs_ was calculated by using the raw data. The list of ideal Bragg-peak angle, 2*θ*_SRM_, was provided in the certificate of SRM 660a^11^. The observed diffraction angle 2*θ*_obs_ for each reflection was chosen at the strongest intensity in the diffraction data near 2*θ*_SRM_. The measurement error of 2*θ*_obs_ was assumed to be the same as the step of 0.008° in the data.

### Data Availability

The data that support the findings of this study are distributed by Prof. Armel Le Bail and available in the website, http://www.cristal.org/powdif/low_fwhm_and_rp.html.
